# Small-Cell Lung Cancer Cavities: Primary or Secondary?

**DOI:** 10.7759/cureus.13691

**Published:** 2021-03-04

**Authors:** Toufic Tannous, Andrew Mak, Matthew Keating

**Affiliations:** 1 Department of Internal Medicine, Roger Williams Medical Center, Providence, USA; 2 Division of Hematology/Oncology, Roger Williams Medical Center, Providence, USA; 3 Division of Hematology/Oncology, University of California, Irvine School of Medicine, Irvine, USA

**Keywords:** small-cell lung cancer, cavitary lesions, aspergillus

## Abstract

Although non-small-cell lung cancer occasionally presents as cavitary lesions, it is rare for small-cell lung cancer (SCLC) to present or progress in such a manner. If a cavitary lesion is seen in the setting of small-cell lung carcinoma, infectious etiologies must be excluded first. We present the case of a 43-year-old man with refractory SCLC that progressed despite two lines of therapy, and who was ultimately found to have recurrent adenopathy and numerous widespread cavitary lung nodules. Fine-needle aspiration of a hilar lymph node revealed extensively necrotic SCLC, while bronchoalveolar cultures grew *Aspergillus fumigatus *and* Candida albicans*. The patient was subsequently treated with voriconazole; however, despite these measures, his overall clinical course deteriorated and the patient ultimately succumbed to his illness. Aspergillosis is a major cause of cavitary lung lesions, especially in immunocompromised patients. Our patient with refractory stage four SCLC was found to have several cavitary lung lesions. Before assuming that cavitary lesions are neoplastic, evaluation for aspergillosis should be conducted, particularly in SCLC patients. Although invasive fungal infections are often missed, it may be prudent to conduct such testing because aspergillosis is a treatable condition and the treatment can improve a patient’s hospitalization and overall clinical course.

## Introduction

A cavitary lung lesion is a gas-filled space within a zone of pulmonary consolidation, mass, or nodule. It is produced by the expulsion of a necrotic part of the lesion via the bronchial tree. The etiology of the cavity can be either infectious or non-infectious. Prominent infectious agents can be bacterial (e.g., *Staphylococcus aureus*, *Klebsiella pneumoniae*, *Haemophilus influenzae*, *Streptococcus pneumoniae*, mycobacterial organisms), fungal (e.g., *Aspergillus*), or parasitic (e.g., *Echinococcus*). Non-infectious etiologies include primary lung tumors, most commonly squamous cell carcinoma or metastatic disease, and on rare occasions, pulmonary emboli [1].

*A. fumigatus* can affect the lung in the following ways: allergic bronchopulmonary aspergillosis, which afflicts patients with long-standing asthma; aspergilloma, which can be found in patients with preexisting lung cavities; chronic necrotizing aspergillosis, affecting patients with chronic lung disease; and invasive aspergillosis, more commonly seen in immunocompromised and critically ill hosts [2]. Lung cancer patients are especially at risk given the high incidence of chemotherapy-induced neutropenia and a compromised immune system.

Regarding primary lung malignancies, non-small-cell lung cancers (NSCLC) have the potential to form cavitary lung lesions or progress in that form. In fact, squamous cell carcinoma is the most common type of NSCLC described that cavitates. In some of these NSCLC cases, aspergilloma is found to be occupying these cavities, and the co-existence of *Aspergillus *and NSCLC is a frequently reported incident [3,4]. However, cases of small-cell lung cancer (SCLC) with concurrent *Aspergillus* and cavitary lesions have only been described previously in rare instances [5].

We describe a patient with extensive-stage refractory SCLC who failed two lines of chemotherapy, progressing to form multiple cavitary lesions. According to our literature search, SCLC does not commonly present or progress as cavitary lung lesions. This anomaly led us to further investigate the nature of these lung lesions and discover a co-existing *Aspergillus* infection.

## Case presentation

A 43-year-old man presented with hoarseness in his voice for five months, fatigue, weakness, and 30-pound weight loss. He had a medical history of essential hypertension treated with beta blockers. He was a 30-pack-year smoker and a chronic alcohol drinker. His family history was pertinent for essential hypertension and diabetes mellitus. A computed tomography (CT) of the chest revealed bulky left mediastinal adenopathy. Subsequent bronchoscopy and transbronchial biopsy of the lesion confirmed the diagnosis of SCLC with thyroid transcription factor 1, synaptophysin, and Ki-67 positive stains. Head CT showed a left paracentral cerebellar mass. The patient initially underwent six cycles of carboplatin and etoposide, and a significant response was seen. He was then given whole-brain radiation and 10 fractions of thoracic radiation.

**Figure 1:**
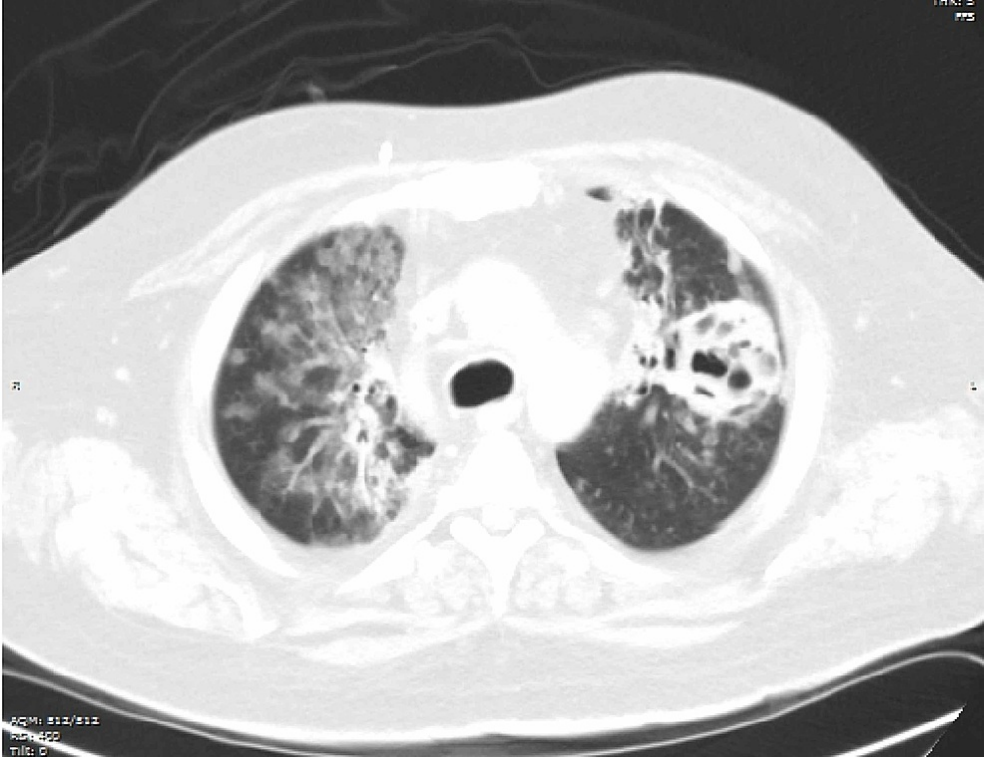
CT scan of the chest without contrast showing pulmonary cavities in a patient with SCLC. CT, computed tomography; SCLC, small cell lung cancer

Repeat CT of the chest/abdomen/pelvis showed a new right paratracheal node and an increase in left hilar adenopathy and a new left adrenal nodule. He was then started on second-line chemotherapy for relapsed/refractory disease with six cycles of irinotecan. Follow-up imaging showed a new cerebellar metastasis and a new right frontal lobe metastasis, as well as an increase in the adrenal nodule size. Due to the cerebral edema seen on imaging, he was started on dexamethasone. After his fatigue improved, his steroids were tapered off and discontinued. He was then scheduled to start third-line therapy with nivolumab, but it was delayed for a few weeks as he developed cholecystitis requiring a cholecystectomy. After the surgery, the patient was recovering well and felt better for a couple of weeks until he started to feel dyspneic again. He was sent to the hospital where a CT chest/abdomen/pelvis showed significant adenopathy and several widespread cavitary lung nodules (Figure 1).

**Figure 2:**
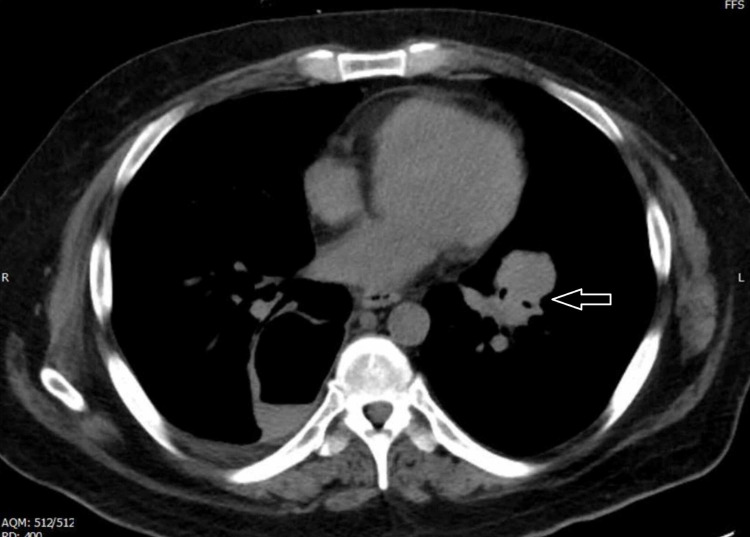
Arrow showing the 4 × 4.2 cm hilar mass that was brushed.

Workup for the cavitary lung lesions included Epstein-Barr virus polymerase chain reaction, anti-streptolysin O antibody titers, anti-DNAse level, and a serum beta-D-glucan level. All results came back within normal limits except for an elevated beta-D-glucan result with a level above 500 pg/mL (reference range: 0-60 pg/mL). A bronchoscopy with a bronchoalveolar lavage (BAL) was performed for all lung lobes. The BAL fluid was sent for gram stain and culture, fungal culture, acid-fast bacilli (AFB), *Legionella*, *Pneumocystis jirovecii*, fluid galactomannan, and cytology. All fungal cultures came back positive for *A. fumigatus* and *Candida albicans*. The left lobe fluid galactomannan yielded a positive result with an index of 2.7 (normal index: <0.5). Brushing of the left hilar mass (Figure 2) came back positive for SCLC with extensive necrosis. The patient was then treated with voriconazole. Unfortunately, due to the patient’s extensive disease and poor prognosis, his overall condition continued to deteriorate despite treatment. He and his family opted for hospice care, and he eventually succumbed to his illness.

## Discussion

This case describes a patient with extensive-stage SCLC who eventually progressed through two lines of chemotherapy and radiotherapy. During his final hospitalization, he was found to have multiple cavitary lung masses and nodules. As it was difficult to elucidate the exact nature of these nodules, bronchoscopy was performed to sample the nearby tissue. Unexpectedly, in the setting of multiple cavitary lesions, the cytology from the left hilar mass showed SCLC with extensive necrosis. As previously mentioned, a cavity is formed after the central necrotic part within a mass is expelled. This is a particularly interesting finding because rare, if any, cases of SCLC with cavities have been reported [1,4]. A few reports have mentioned the cavitation of SCLC lesions, but this was mostly seen after treatment with anti-angiogenic agents, which was not the case here [6]. Moreover, BAL cultures grew *A. fumigatus*, and BAL fluid was positive for galactomannan. This also confounds the clinical scenario as the origin of these nodules could be small-cell tumors, invasive aspergillosis, or a combination of both with small-cell lung cavitations containing aspergillomas. It is very difficult to distinguish invasive aspergillosis solely based on imaging [3].

Most cases of primary cavitary lung lesions are non-small cell in origin, mainly squamous cell carcinoma and adenocarcinoma [4]. These cavitating NSCLC lesions tend to overexpress the epidermal growth factor receptor marker [5]. The reason behind this characteristic association could be secondary to the tumor type itself or due to previous therapy [7]. In fact, around 19% of NSCLC lesions are thought to cavitate after treatment with anti-angiogenic therapy [8]. It was previously considered that cavitary lesions represent chemo-resistant lesions and may carry a worse outcome than non-cavitary lesions [9]. So far, the prognostic significance of such a presentation remains to be seen. Multiple studies have been performed to elucidate any prognostic implications, but none has yielded results thus far [10,11]. Other studies tried to associate wall thickness with outcome and chemo-responsiveness [12]. To date, the clinical relevance and prognostic implications remain unknown.

*A. fumigatus* is one of the several infectious causes of lung cavities and can present in the lung in various ways depending on the patient’s underlying pulmonary disease, presence of organ failure, and current immune status on or off immunosuppressive agents [13]. Invasive aspergillosis is seen in patients with a compromised immune system, causing cavitary lung lesions. It can mimic metastatic lung disease [14], or it can coexist within the primary lung cancer [15]. In many cases, this phenomenon has also been seen after NSCLC treatment, including chemotherapy or surgery [16,17]. Some studies have shown that surface binding proteins such as E-cadherins may play a role in the affinity between non-small-cell tumor cells and the *Aspergillus *species [18].

The association between aspergillosis and SCLC has not been documented extensively, and the presence of *Aspergillus* in our patient may appear to be an unusual finding. However, it is quite possible that *Aspergillus* infections are frequently overlooked in SCLC versus NSCLC patients. There are nearly six-fold as many cases of NSCLC as there are of SCLC in the United States [19]. SCLC almost invariably recurs and is such a threat to mortality that it is easy to ignore how other causes contribute to a patient’s decline. We would argue that, given the data available and cases like ours, a heightened awareness of *Aspergillus* in SCLC is indicated, particularly when ambiguous cavitating lesions are present.

Based on NSCLC’s predilection for cavitation, one might assume that there would be a higher incidence of *Aspergillus* infections in NSCLC versus SCLC patients. However, a retrospective study discredits this assumption, and we would like to draw attention to this study to emphasize the importance of increased awareness for *Aspergillus* infections in SCLC patients. In a retrospective analysis of 1,711 lung cancer patients, 2.63% were found to have developed invasive pulmonary aspergillosis. NSCLC patients greatly outnumbered SCLC patients in the study. However, when the aspergillosis infection rate was broken down by lung cancer subtype, the results were comparable in NSCLC versus SCLC, with no significant difference between the two groups (2.51% in NSCLC versus 3.28% in SCLC) [16].

Considering the high incidence of chemotherapy-induced neutropenia and prolonged steroid use in SCLC, it is not a surprise that bacterial, viral, and/or fungal infections can complicate treatment. Furthermore, with the rise of immunotherapy use in extended-stage SCLC, high-dose steroids often provide the antidote to immunotherapy side effects but also predispose patients to more infections. This raises the question of prophylactic antifungal therapy in these patients. Antifungal therapy is the mainstay for prophylaxis against invasive fungal infections in post-transplant leukemia/lymphoma patients. For practical purposes, any cancer patient on prolonged steroids and chemotherapy can be considered for antifungal prophylaxis. Both prolonged steroids and chemotherapy administration in the month preceding infection are significant risk factors for invasive pulmonary aspergillosis in lung cancer patients [16]. However, several trials have failed to show a mortality benefit for antifungal prophylaxis in non-transplant patients [20]. Without a strict guideline for antifungal prophylaxis in solid tumor patients, this puts an even greater emphasis on the importance of expedient detection and treatment of invasive fungal infections.

## Conclusions

Testing cavitary lesions for the presence of *A. fumigatus* in the setting of SCLC or NSCLC is not routinely done but should be performed if such lesions emerge in these patients. Infectious etiologies should be considered in addition to disease recurrence. Treatment for aspergillosis is widely available and can greatly improve a patient’s clinical outcome if implemented early. Nearly all SCLC patients eventually have disease relapse, but forgoing further workup for abundant cavitary lesions can do the patient a great disservice.
